# The Burden of Influenza and RSV among Inpatients and Outpatients in Rural Western Kenya, 2009–2012

**DOI:** 10.1371/journal.pone.0105543

**Published:** 2014-08-18

**Authors:** Gideon O. Emukule, Sammy Khagayi, Meredith L. McMorrow, Rachel Ochola, Nancy Otieno, Marc-Alain Widdowson, Melvin Ochieng, Daniel R. Feikin, Mark A. Katz, Joshua A. Mott

**Affiliations:** 1 Centers for Disease Control and Prevention (CDC)-Kenya Country Office, Nairobi, Kenya; 2 Kenya Medical Research Institute (KEMRI), Nairobi and Kisumu, Kenya; 3 Influenza Division, National Center for Immunization and Respiratory Diseases, US Centers for Disease Control and Prevention, Atlanta, Georgia, United States of America; 4 Division of Preparedness and Emerging Infections, Centers for Disease Control and Prevention, Atlanta, Georgia, United States of America; 5 Centers for Disease Control and Prevention, Port-au-Prince, Haiti; 6 United States Public Health Service, Rockville, Maryland, United States of America; University of Texas Medical Branch, United States of America

## Abstract

**Background:**

In Kenya, detailed data on the age-specific burden of influenza and RSV are essential to inform use of limited vaccination and treatment resources.

**Methods:**

We analyzed surveillance data from August 2009 to July 2012 for hospitalized severe acute respiratory illness (SARI) and outpatient influenza-like illness (ILI) at two health facilities in western Kenya to estimate the burden of influenza and respiratory syncytial virus (RSV). Incidence rates were estimated by dividing the number of cases with laboratory-confirmed virus infections by the mid-year population. Rates were adjusted for healthcare-seeking behavior, and to account for patients who met the SARI/ILI case definitions but were not tested.

**Results:**

The average annual incidence of influenza-associated SARI hospitalization per 1,000 persons was 2.7 (95% CI 1.8–3.9) among children <5 years and 0.3 (95% CI 0.2–0.4) among persons ≥5 years; for RSV-associated SARI hospitalization, it was 5.2 (95% CI 4.0–6.8) among children <5 years and 0.1 (95% CI 0.0–0.2) among persons ≥5 years. The incidence of influenza-associated medically-attended ILI per 1,000 was 24.0 (95% CI 16.6–34.7) among children <5 years and 3.8 (95% CI 2.6–5.7) among persons ≥5 years. The incidence of RSV-associated medically-attended ILI was 24.6 (95% CI 17.0–35.4) among children <5 years and 0.8 (95% CI 0.3–1.9) among persons ≥5 years.

**Conclusions:**

Influenza and RSV both exact an important burden in children. This highlights the possible value of influenza vaccines, and future RSV vaccines, for Kenyan children.

## Introduction

Acute lower respiratory tract infections account for an estimated 1.9 million deaths in children <5 years of age annually, up to 90% of which occur in the developing world [1]–[3]. In Africa and Asia, acute lower respiratory tract infections are two to ten times more common than in the USA [4], and have three to seven-fold worse outcomes than in the industrialized world [5]. A large fraction of human respiratory tract infections are associated with viruses including influenza A and B, and respiratory syncytial virus (RSV) [6]. Safe and effective vaccines for influenza are available [7], [8], but very limited quantities of influenza vaccine are currently being used in Kenya, and elsewhere in Africa [9]. Efforts are on-going to develop a vaccine for RSV [10]. However additional and more detailed data on burden of disease may be required before these vaccines are used more widely.

While some data suggest a significant burden of hospitalized influenza in Kenya, many of the data come from persons seeking medical care during the period of the 2009 influenza A(H1N1)pdm09 pandemic with few data from either the pre- or post-pandemic periods [11]–[13]. The age-specific burden of influenza has also not clearly been defined among infants <6 months, or among those aged 6–12 months. Such data could be used to inform whether vaccination of pregnant women to protect infants <6 months of age (for whom no influenza vaccine is currently licensed) through maternal antibody transfer and/or vaccination of children older than six months of age may be viable vaccination strategies for Kenya. Data on the burden of RSV have also been limited to those seeking medical care – focused on children <5 years – at the Coastal and Western areas of Kenya [14]–[17].

To address these gaps we estimated the age-specific burden and seasonality of medically-attended, and non-medically attended influenza and RSV in western Kenya during the period 2009–2012.

## Methods

Since August 2009 the Kenya Medical Research Institute and the Centers for Disease Control and Prevention (KEMRI/CDC) have conducted hospital-based surveillance for severe acute respiratory illness (SARI) at Siaya District Hospital (SDH) and outpatient surveillance for influenza-like illness (ILI) at Ting'wang'i Health Centre (THC) in western Kenya. Both facilities are located in Karemo Division, where KEMRI/CDC implements a Health Demographic Surveillance System (HDSS), with an approximate population of 80,000 [18]. Karemo's population is culturally homogenous, and almost entirely rural [19]. The area is endemic for malaria with a high child mortality rate (212 deaths per 1000 live births in 2008) [19] and a high HIV prevalence, which in 2008 was estimated at 14% among persons aged ≥18 years [20].

### Data collection and case definitions

From August 2009 to July 2012, trained nurses and clinical officers enrolled all consenting patients who were admitted to SDH with SARI or sought outpatient healthcare at THC for ILI. ILI was defined as axillary temperature ≥38°C and cough or sore throat with an onset with the past 14 days in an outpatient for persons of all ages. SARI was defined as hospitalization with cough or difficulty breathing or pleural chest pain with an onset within the last 14 days. Hospitalized SARI patients were followed up for the duration of their hospitalization until they were discharged, transferred or died. We also estimated rates of non-medically attended ILI and SARI using the proportion of persons that sought care for acute respiratory infections (ARI), and pneumonia, respectively, from a health utilization survey implemented in Western Kenya in 2005 [21]. In this survey, ARI was defined more narrowly than ILI as cough and difficulty in breathing within the last 14 days [21]. Pneumonia was defined as ARI lasting for more than two days (excluding the past 14 days) within the preceding 12 months or a diagnosis of 'pneumonia' by a healthcare worker. Data processing and management procedures have been described previously [13].

### Specimen collection and laboratory methods

Patients meeting SARI and ILI case definitions were enrolled in medical facility-based surveillance and nasopharyngeal (NP) and oropharyngeal (OP) swabs were collected, combined into a single viral transport media, and tested by real-time reverse transcription polymerase chain reaction (rRT-PCR) for influenza A and B viruses and RSV [12], [13].

Patients admitted to the hospital for any reason were offered voluntary counseling and testing for HIV. Details of HIV testing at SDH have also been described previously [22].

### Data analysis

#### Descriptive analyses

We used univariate analysis methods including mean, median and proportions to describe demographic characteristics and laboratory outcomes of patients. Chi-square tests were used to assess associations for categorical variables. Mann-Whitney rank sum tests were used to test for differences in the age distribution between patients who were tested and those who were not tested for influenza viruses and RSV.

#### Estimating the incidence rates of hospitalized SARI, and influenza and RSV-associated SARI

**Figure 1:**
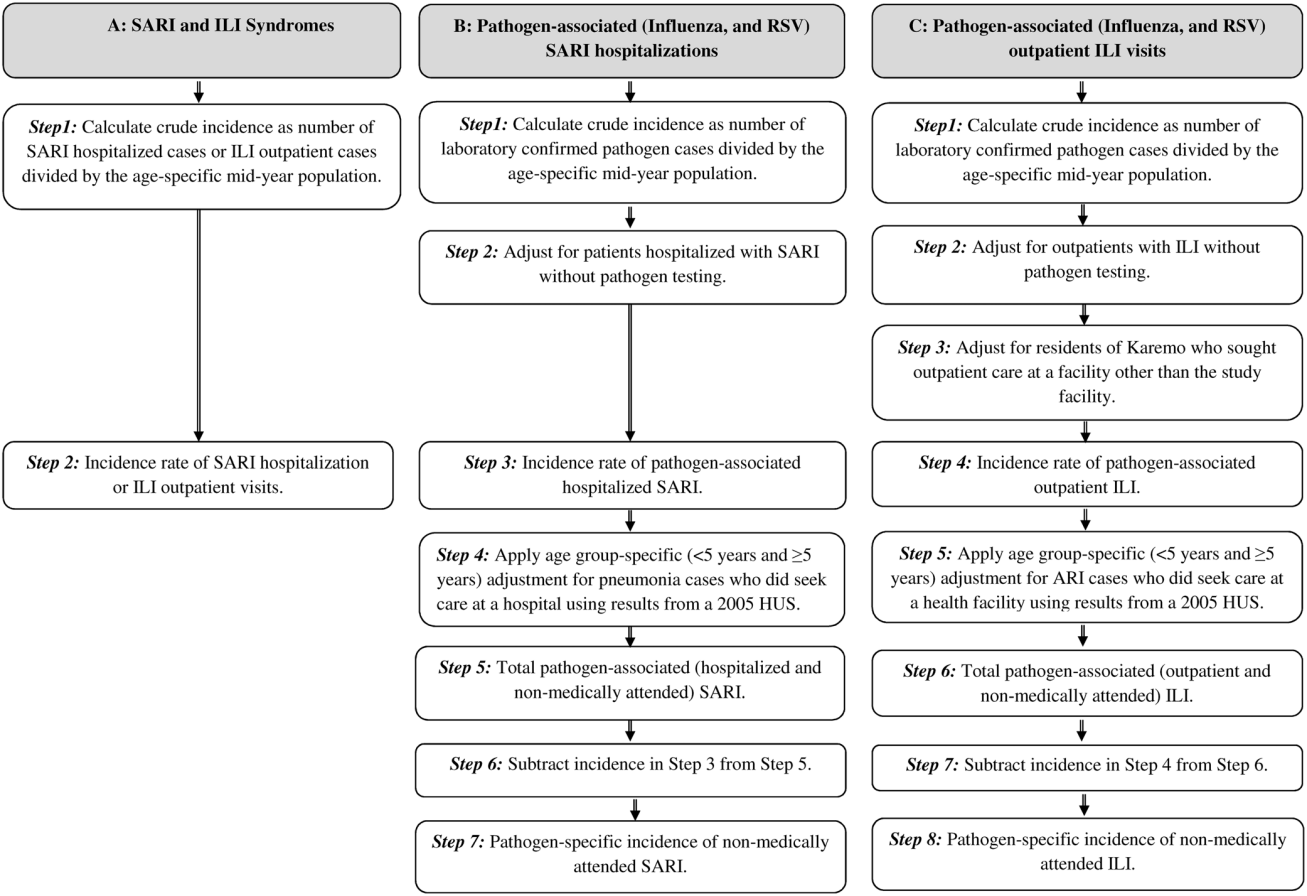
Flow diagram of the steps used for the calculation of incidence rates.

As SDH is the only hospital located within Karemo Division, age-specific incidence rates (for 11 age groups: <6 months, 6–11 months, 12–23 months, 2–4 years, 5–17 years, 18–34 years, 35–49 years, ≥50 years, <5 years, ≥5 years, all ages) of hospitalized SARI were estimated by dividing the number of cases enrolled in the HDSS for each age group, by the mid-year population in each age group residing in Karemo Division who were enrolled in HDSS. Similarly, the age-specific incidence rates of Influenza and RSV-associated SARI hospitalizations were calculated by dividing the number laboratory-confirmed influenza or RSV cases enrolled in the HDSS for each age group, by the mid-year population in each age group. Rates of influenza and RSV-associated SARI were adjusted for patients who met the SARI case definition but from whom NP/OP swabs were not collected and tested (for each age group, the adjusted number of cases was equal to the number of laboratory-confirmed cases divided by the proportion of patients with SARI who were tested). We compared incidence rates of influenza and RSV for residents living within 0–5 kilometers (KM), 0–10 KM, and 0–20 KM of the hospital. We did this in order to assess whether rates of hospitalized influenza and RSV declined with inclusion of persons who lived within Karemo Division but farther from the hospital (Figure 1).

#### Estimating the incidence rates of outpatient ILI, and Influenza and RSV-associated ILI

We estimated age-specific incidence of outpatient influenza and RSV virus infections for the 11 aforementioned age groups. To do this we examined the outpatient registers for children <5 years and persons ≥5 years at all seven outpatient facilities in Karemo Division to obtain the area of residence (village and sub-location) for each outpatient visit for a two-year period. From each outpatient facility we calculated the number and proportion of patients seen who were residents of Karemo Division by month and age (<5 years and ≥5 years). We summed the number of patients seen at the seven facilities who were residents of Karemo and calculated the proportion of those who visited THC. We determined that approximately 10% (12,464/128,967) of outpatient visits among Karemo residents between 2010 and 2011 occurred at THC (11% and 9% for children <5 years and persons ≥5 years, respectively). The crude incidence rates (outpatient medically-attended ILI; influenza-associated ILI; and RSV-associated ILI) were calculated by dividing the number of cases by the mid-year population in each age group residing in the Karemo Division who were enrolled in HDSS. After calculating crude rates by age group, we calculated adjusted rates by dividing the crude rates by the proportions of patients who visited THC in each age group (0.11 for children <5 years and 0.09 for persons ≥5 years). Influenza and RSV-associated incidence rates were then further adjusted for ILI patients who did not have NP/OP swabs collected (for each age group, the adjusted number of cases was equal to the number of laboratory-confirmed cases divided by the proportion of patients with ILI who were tested) (Figure 1).

#### Estimating non-medically attended incidence rates of influenza and RSV

We calculated the total (hospitalized and non-hospitalized) incidence of influenza and RSV-associated SARI by dividing the hospitalized SARI-associated incidence rates by the proportion of reported cases of pneumonia who went to hospital based on data from a 2005 Health Utilization Survey. In this survey, health utilization was reported for two age groups – children <5 years and persons ≥5 years. Forty eight percent (95% CI 35–62) of the children aged <5 years and 34% (95% CI 23–48) of persons aged ≥5 years were reported to have sought care for pneumonia at a hospital. As there was no health utilization reported for the finer age categories among children <5 years and persons ≥5 years, we used the same adjustment factor reported in the survey for children <5 years (48%) for all the age categories under five years (<6 months, 6–11 months, 12–23 months, 2–4 years, and <5 years). Similarly, we used the same health utilization adjustment factor (34%) reported for persons ≥5 years for all the age groups among persons aged five years or older (5–18 years, ≥18 years, and ≥5 years). The incidence of non-hospitalized SARI associated with influenza and RSV was then calculated by subtracting the hospitalized influenza and RSV-associated SARI incidence from the total incidence of influenza and RSV-associated SARI calculated using health utilization survey data for each age group (Figure 1).

We took a similar approach to estimate non-medically attended ILI associated with influenza and RSV. In this survey, 42% (95% CI 33–51) of the children aged <5 years and 44% (95% CI 40–53) of persons aged ≥5 years reported to have sought care for ARI at a health facility. We used an adjustment factor of 42% for all the age groups under the age of five years and 44% for the age groups among patients aged five years or older. Total age-specific incidence rates of influenza and RSV-associated ILI were calculated by dividing the medically-attended ILI incidence rates by the proportion of reported cases of ARI in the health utilization survey who went to any facility. The age-specific incidence of non-medically attended ILI associated with influenza and RSV was calculated by subtracting the adjusted incidence of medically-attended influenza and RSV estimated from ILI surveillance at THC from the total incidence of influenza and RSV-associated ILI calculated using health utilization survey data, for each age group (Figure 1). Incidence rates were reported per 1000 persons, and the Poisson approximation method was used to calculate the 95% confidence intervals (CIs) around point estimates [23], separately for each age group. Data analysis was performed using Stata version 12.1 (Stata Corp, College Station, Texas).

### Ethical review

This study was approved by both the Ethical Review Committee of the Kenya Medical Research Institute (KEMRI SSC-1801) and Institutional Review Board of CDC-Atlanta (CDC IRB #3308). Written informed consent was obtained from all participants or caretakers/guardians of all minors prior to enrolment in the study and sample collection.

## Results

### Descriptive analyses

We enrolled 5507 patients hospitalized with SARI at SDH, and 1632 patients with ILI at THC. Most (SARI = 75%, ILI = 77%) were children <5 years old. Although the study case definition was different from the current WHO case definition for SARI, seventy percent of the SARI cases included in this study also met the new WHO case definition [75% among children <5 years (70% in children <6 months); and 53% in persons ≥5 years] [24]. The median age was 1.6 years and 2.4 years for SARI and ILI patients, respectively, and 50% of the SARI patients and 53% of the ILI patients were female. Of the enrolled patients, 4387 (80%) of those with SARI and 1508 (92%) of those with ILI were tested for influenza and RSV (Table 1). When we compared SARI patients who were tested with those who were not, those tested were more likely to be male (50% vs. 46%; p<0.05), and the median age among those tested was 1.6 years compared to 1.5 years among those not tested (p<0.001). Similarly, ILI patients who were tested for influenza viruses or RSV were significantly older than those not tested (median age 2.4 years vs. 1.8 years; p<0.001). Case-fatality was significantly higher in SARI patients who were not tested for influenza or RSV than in those who were tested (18% vs. 4%; p<0.001).

**Table 1:** Characteristics of patients who were admitted at SDH with SARI and those who presented to THC with ILI and were tested for respiratory pathogens, August 2009– July 2012.

	In-patients (SARI patients)		Out-patients (ILI patients)	
	Total Number (N = 5,507)	Enrolled in Karemo HDSS (N = 2,136)	Total Number (N = 1,632)	Enrolled in Karemo HDSS (N = 1,186)
	n (%)	n (%)	n (%)	n (%)
**Sex**				
Male	2,800(50.8)	1,097(51.4)	779(47.7)	567(47.8)
Female	2,707(49.2)	1,039(48.6)	853(52.3)	619(52.2)
**Age Group**				
<6 months	918(16.7)	351(16.4)	134(8.2)	85(7.2)
6–11 months	1,040(18.9)	414(19.4)	207(12.7)	140(11.8)
12–23 months	1,134(20.6)	495(23.2)	339(20.8)	248(20.9)
2–4 years	1,018(18.5)	411(19.2)	570(34.9)	431(36.3)
5–17 years	417(7.6)	174(8.2)	311(19.1)	236(19.9)
18–34 years	493(9.0)	133(6.2)	53(3.3)	35(3.0)
35–49 years	223(4.1)	74(3.5)	11(0.7)	7(0.6)
≥50 years	264(4.8)	84(3.9)	7(0.4)	4(0.3)
Median age years (Range)	1.6(0.01–90.0)	1.6(0.01–90.0)	2.4(0.1–0 63.4)	2.4(0.02–63.4)
**Died in the hospital**	390(7.1)	103(4.8)	-	-
**HIV status**				
Negative	2,917(53.0)	1,190(55.7)	482(29.5)	337(28.4)
Positive¥	654(11.9)	230(10.8)	28(1.7)	12(1.0)
Unknown	1,936(35.2)	716(33.5)	1,122(68.8)	837(70.6)
**Sampled and tested for influenza and RSV**	4,387(79.7)	1730(81.0)	1,508(92.4)	1,092(92.1)
**Respiratory pathogen isolated**				
Influenza A or B*	348(7.9)	124(7.2)	206(13.7)	155(14.2)
Influenza A*	253(5.8)	93(5.4)	159(10.5)	119(10.9)
Seasonal A (H1N1)**	*2(0.8)*	*0(0.0)*	*1(0.6)*	*1(0.8)*
Seasonal A (H3N2)**	*63(24.9)*	*16(17.2)*	*52(32.7)*	*42(35.3)*
Pandemic H1N1**	*93(36.8)*	*39(41.9)*	*82(51.6)*	*59(49.6)*
Unsubtypable**	*10(4.0)*	*3(3.2)*	*3(1.9)*	*3(2.5)*
Not subtyped**	*81(32.0)*	*32(34.4)*	*18(11.3)*	*14(11.8)*
Influenza B*	97(2.2)	32(1.9)	51(3.4)	38(3.5)
Influenza A and B*	2(0.1)	1(0.1)	4(0.3)	2(0.2)
Respiratory syncytial virus (RSV)*	437(10.0)	176(10.2)	138(9.7)	101(9.7)
Influenza-RSV co-infection*	18(0.4)	7(0.4)	14(0.9)	11(1.0)

¥ 18% of those with known HIV status were HIV infected; *Denominator is the number of those who tested for Influenza and RSV; **Denominator in the number of influenza A cases.

Influenza viruses were detected in 348/4387 (8%) of the SARI patients (Table 1); 253 (6%) were positive for influenza A, 97 (2%) were positive for influenza B and 2 (0.1%) were positive for positive for both influenza A and B. The most commonly detected subtypes during the study period were influenza A(H1N1)pdm09 and influenza A(H3N2). RSV was detected in 437 (10%) of the SARI patients who were tested (12% in children <5 years; 4% in persons ≥5 years old). HIV-infected patients were as likely as non-HIV infected patients to test positive for these viral pathogens when we adjusted for age (p = 0.284 for influenza; and p =  0.957 for RSV). The case-fatality proportion was similar for SARI patients who tested positive compared to those who tested negative for influenza [13/348 (4%) vs. 171/4039 (4%); p = 0.66] and RSV [14/437 (3%) vs. 170/3950 (4%); p = 0.28], respectively. The median age among the SARI patients was significantly higher in influenza positive patients compared to RSV positive patients [3.0 vs. 1.1 years; p<0.001].

Of the 1508 ILI patients tested, 206 (14%) tested positive for influenza viruses; [159 (11%) were positive for influenza A; 51 (3%) for influenza B; and 4 (0.3%) were positive for both influenza A and B]. The most commonly detected subtypes during the three years were influenza A(H1N1)pdm09 and influenza A(H3N2). RSV was detected in 138 out of 1,508 patients (10%) [11% in children <5 years; 6% in persons ≥5 years old] (Table 1). Similarly, the median age among ILI patients was significantly higher in the influenza positive patients compared to RSV positive patients [4.4 vs. 2.4; p<0.001].

**Figure 2:**
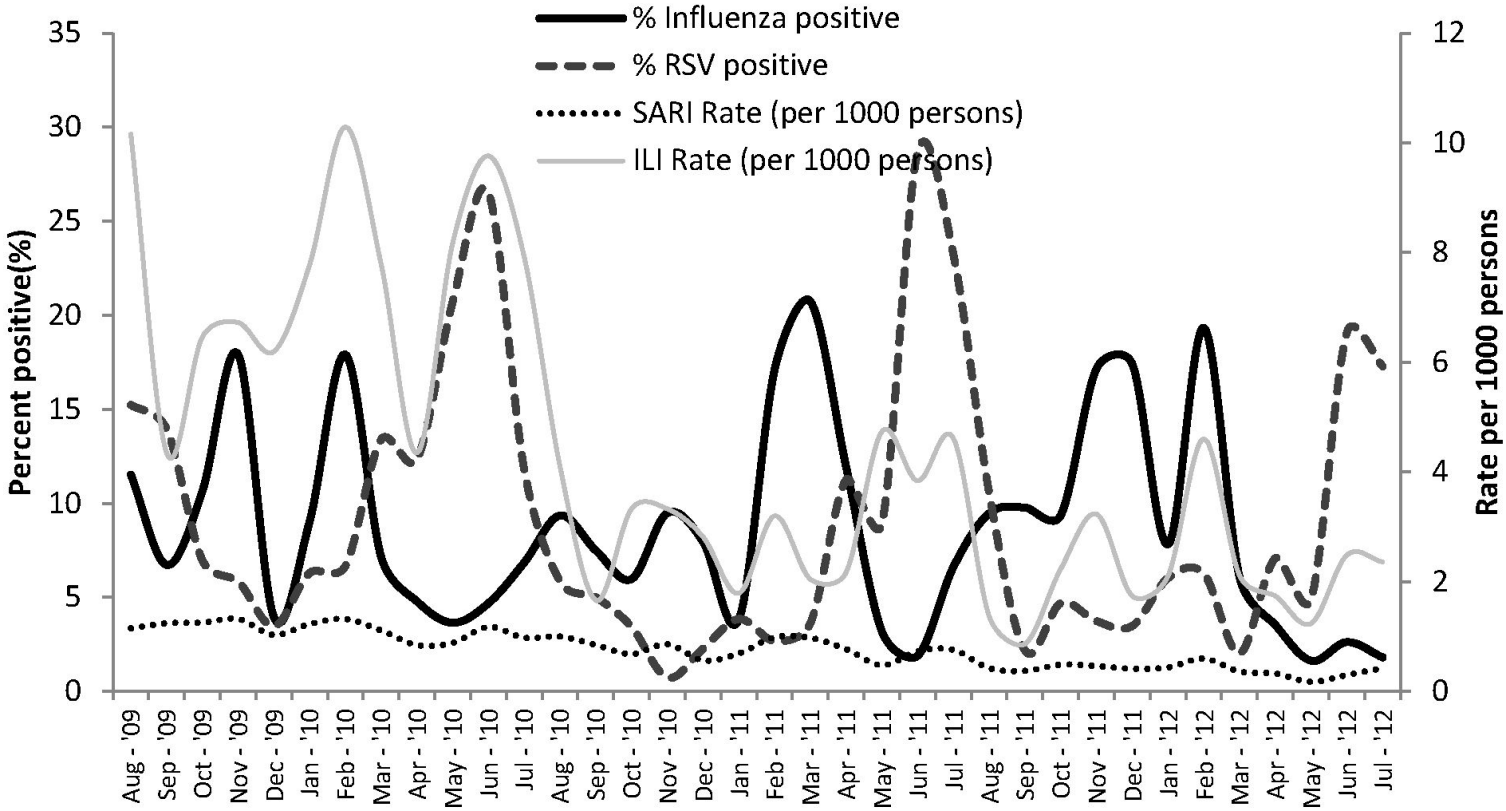
Seasonality of Influenza and RSV in Western Kenya, Aug 2009– Jul 2012.

**Figure 3:**
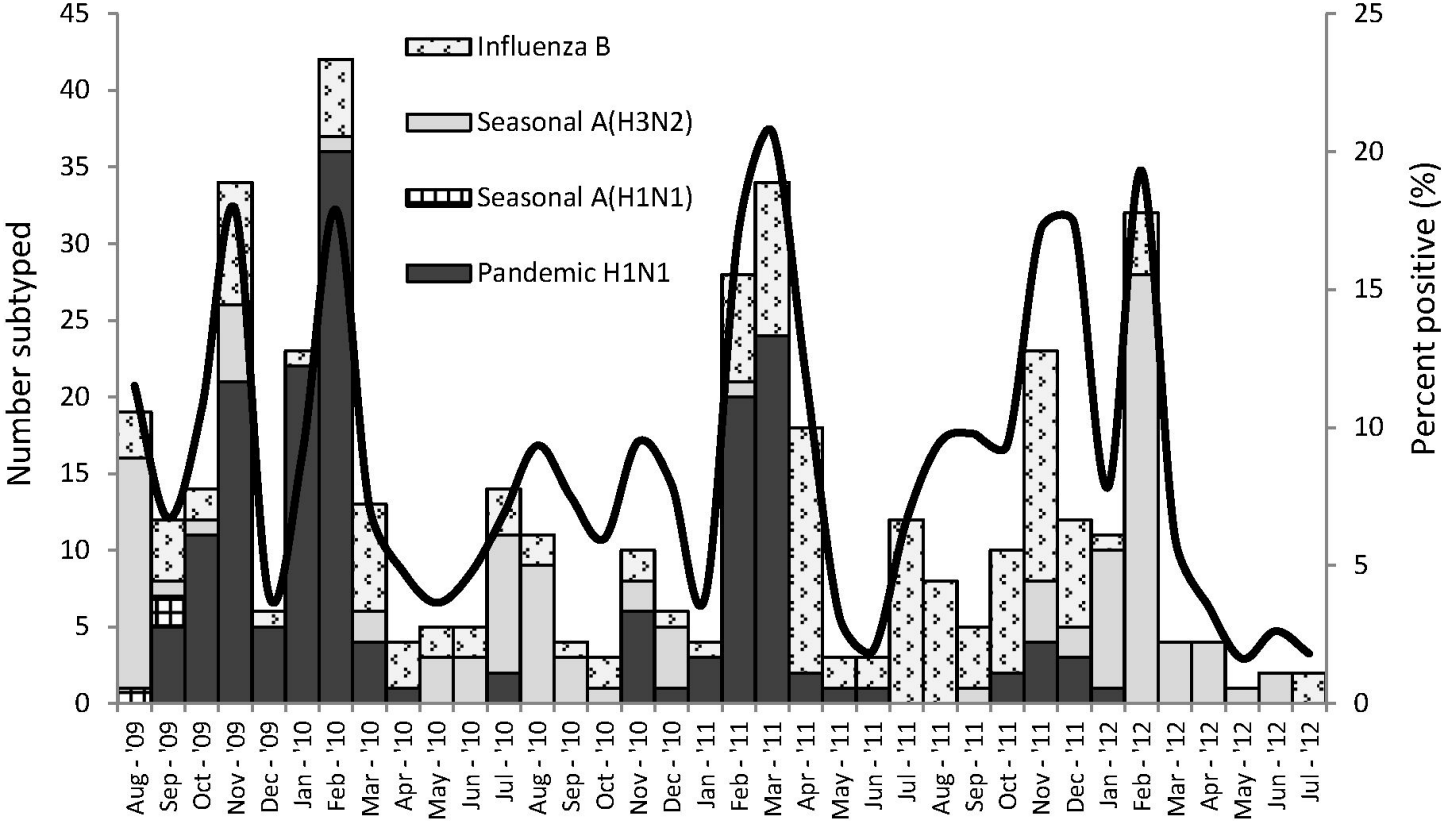
Seasonality of influenza and circulating subtypes in Western Kenya, Aug 2009– Jul 2012.

Influenza-virus-positive cases were detected throughout the study period with the influenza-positive proportions observed to be lower in the hotter months between December and January every year. RSV peaked more predictably during May-July of each year (Figure 2). Influenza A(H1N1)pdm09 was the dominant influenza virus in circulation during late 2009 and early 2010, and again during late 2010 and early 2011. Influenza A(H3N2) was the dominant influenza virus in circulation during the periods May 2010 to October 2010 and also from January 2012 to July 2012. Influenza B viruses were detected mostly between April 2011 and December 2011 (Figure 3).

### Incidence of hospitalized SARI, and influenza and RSV-associated SARI

**Figure 4:**
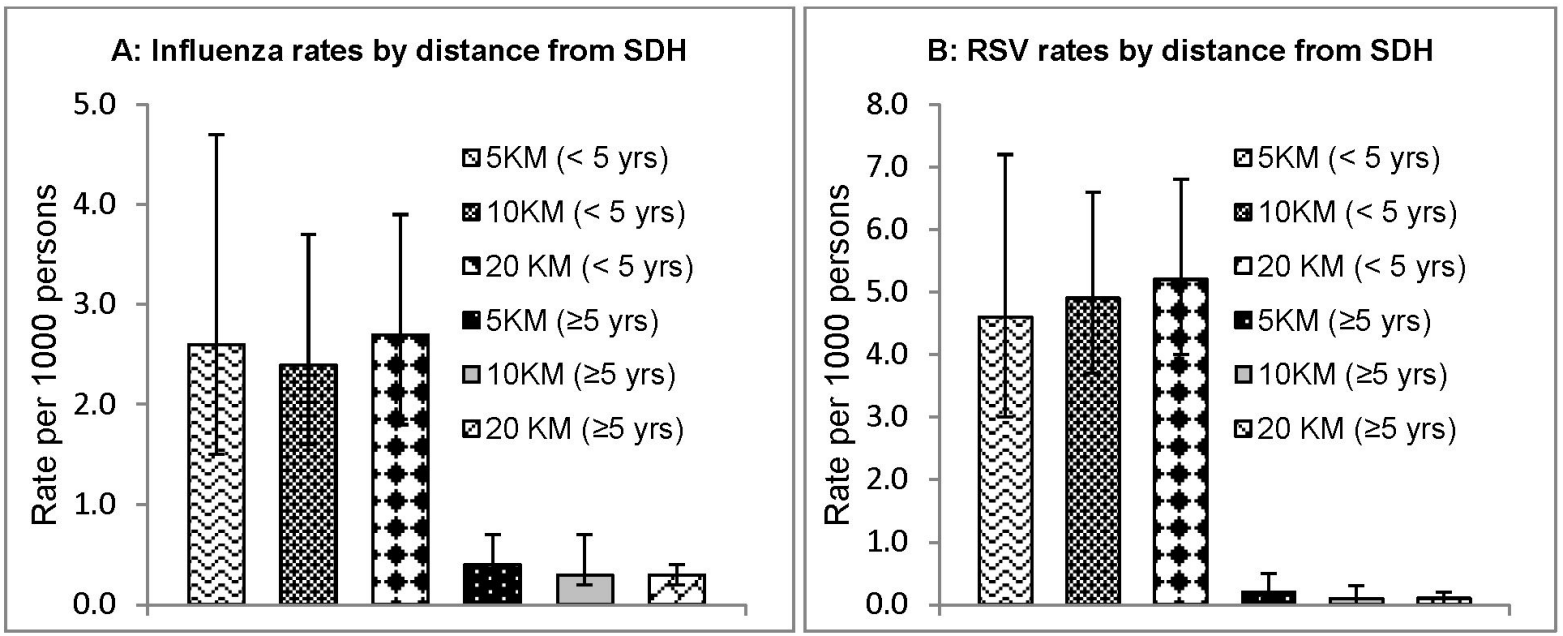
Rates of influenza and RSV associated SARI by distance from SDH, Aug 2009– Jul 2012.

The estimated average annual incidence rate of SARI hospitalizations among children <5 years of age was 43.6(95% CI 40.1–47.3) [106.2(95% CI 88.6–127.3) in children <6 months; 120.2(95% CI 101.7–142.0) in children 6–11 months; 67.6(95% CI 58.1–78.8) in children 12–23 months; and 16.9(95% CI 14.3–20.0) in children 2–4 years] and 2.4(95% CI 2.0–2.8) among persons aged ≥5 years cases per 1000 persons (Table 2). The average annual adjusted incidence of influenza-associated hospitalized SARI was 0.7 per 1000 persons (95% CI 0.5–0.9) and was higher among children aged <5 years [2.7 (95% CI 1.8–3.9)] compared to persons aged ≥5 years [0.3 (95% CI 0.2–0.4)] (Table 3). Influenza-associated hospitalizations were 5.7(95% CI 2.4–13.8) in children <6 months; 4.7(95% CI 1.8–11.9) in children aged 6–11 months; and 4.4(95% CI 2.3–8.5) in children aged 12–23 months. The incidence of influenza-associated SARI hospitalizations was not significantly different among residents of Karemo living within 5 KM of SDH [<5 years = 2.6 (95% CI 1.5–4.7); ≥5 years = 0.4(95% CI 0.2–0.7)] compared to residents living within 10 KM of SDH [<5 years = 2.4 (95% CI 1.6–3.7); ≥5 years = 0.3(95% CI 0.2–0.5)] and those living with 20 KM of SDH [<5 years = 2.7 (95% CI 1.8–3.9); ≥5 years = 0.3(95% CI 0.2–0.4)] (Figure 4).

**Table 2:** Age-specific average annual rates of SARI hospitalizations and ILI outpatient visits (per 1,000 persons) in Western Kenya, August 2009– July 2012.

	Karemo Population^a^	Proportion of hospitalizations^b^ with SARI n(%)	Average annual SARI hospitalization rates (95% CI)	Proportion of outpatients^c^ with ILI n(%)	Average annual medically-attended ILI rates^d^ (95% CI)
**Age Group**					
<6 months	1,102	351/821(42.8)	106.2(88.6–127.3)	85/896(9.5)	233.7(206.9–264.1)
6–11 months	1,148	414/1,050(39.4)	120.2(101.7–142.0)	140/858(16.3)	369.5(336.0–406.4)
12–23 months	2,440	495/1,287(38.5)	67.6(58.1–78.8)	249/1,436(17.3)	309.2(287.9–332.1)
2–4 years	8,097	411/1,267(32.4)	16.9(14.3–20.0)	433/2,435(17.8)	162.1(153.5–171.1)
5–17 years	28,326	174/708(24.6)	2.0(1.6–2.6)	237/2,456(9.6)	31.0(29.0–33.1)
18–34 years	18,270	133/1,452(9.2)	2.4(1.8–3.3)	35/1,152(3.0)	7.1(6.0–8.4)
35–49 years	8,272	74/758(9.8)	3.0(2.0–4.4)	7/461(1.5)	3.1(2.1–4.6)
≥50 years	10,397	84/944(8.9)	2.7(1.9–3.9)	4/749(0.5)	1.4(0.9–2.4)
<5 years	12,787	1,671/4,425(37.8)	43.6(40.1–47.3)	907/5,625(16.1)	214.9(207.1–223.1)
≥5 years	65,265	465/3,862(12.0)	2.4(2.0–2.8)	283/4,818(5.9)	16.1(15.1–17.1)
** All ages**	**78,052**	**1,978/8,287(23.9)**	**8.4(7.8–9.1)**	**1,179/10,443(11.3)**	**50.4(48.8–52.0)**

a Mid–study-period population for Karemo (Aug 2009– Jul 2012); ^b^Total hospitalizations among residents of Keremo over three years (Aug 2009– Jul 2012); ^c^Total outpatient visits to Ting'wang'i by residents of Karemo over three years (Aug 2009– Jul 2012); ^d^Adjusted for outpatient visits to other health facilities in Karemo other than Ting‘wang’i.

**Table 3:** Age-specific average annual rates for hospitalizations and outpatient visits attributable to influenza and RSV (per 1,000 persons) in Western Kenya, August 2009– July 2012^a^.

	SARI				ILI			
	Proportion positive^b^ n(%)	Unadjusted hospitalized rates (95% CI)	Adjusted Hospitalized rates^c^ (95% CI)	Non-hospitalized rates^d^ (95% CI)	Proportion positive^b^ n(%)	Unadjusted medically-attended rates (95% CI)	Adjusted Medically-attended rates^c^ (95% CI)	Non-medically attended rates^e^ (95% CI)
**Influenza A or B**								
<6 months	15/279(5.4)	4.5(1.9–10.9)	5.7(2.4–13.8)	6.2(2.9–13.2)	5/72(6.9)	1.5(0.3–6.9)	16.2(3.5–73.8)	22.3(15.0–33.2)
6–11 months	13/337(3.9)	3.8(1.5–9.7)	4.7(1.8–11.9)	5.0(2.2–11.4)	13/128(10.2)	3.8(1.5–9.7)	37.7(14.7–96.7)	52.1(40.4–67.1)
12–23 months	27/409(6.6)	3.7(1.9–7.1)	4.4(2.3–8.5)	4.8(2.7–8.5)	23/223(10.3)	3.1(1.5–6.4)	31.7(15.6–64.4)	43.8(36.3–53.0)
2–4 years	27/319(8.5)	1.1(0.6–2.1)	1.4(0.7–2.7)	1.5(0.9–2.7)	43/402(10.7)	1.8(1.1–3.0)	17.3(10.3–29.0)	23.9(20.8–27.5)
5–17 years	13/126(10.3)	0.2(0.1–0.4)	0.2(0.1–0.5)	0.4(0.2–0.7)	54/221(24.4)	0.6(0.4–1.0)	6.1(3.9–9.8)	7.8(6.9–8.9)
18–34 years	15/117(12.8)	0.3(0.1–0.7)	0.3(0.1–0.7)	0.6(0.3–1.1)	12/35(34.3)	0.2(0.1–0.6)	2.0(0.7–5.3)	2.5(1.9–3.4)
35–49 years	8/67(11.9)	0.3(0.1–1.1)	0.4(0.1–1.2)	0.7(0.3–1.6)	*	*	*	*
≥50 years	6/76(7.9)	0.2(0.0–0.8)	0.2(0.1–0.9)	0.4(0.2–1.1)	*	*	*	*
<5 years	82/1344(6.1)	2.1(1.5–3.1)	2.7(1.8–3.9)	2.9(2.1–4.0)	84/825(10.2)	2.2(1.5–3.2)	21.8(15.1–31.6)	30.1(27.3–33.3)
≥5 years	42/386(10.9)	0.2(0.1–0.4)	0.3(0.2–0.4)	0.5(0.4–0.7)	71/267(26.6)	0.4(0.2–0.5)	4.3(2.8–6.4)	5.4(4.9–6.0)
** All ages**	**124/1730(7.2)**	**0.5(0.4–0.7)**	**0.7(0.5–0.9)**	**1.2(0.9–1.4)**	**155/1092(14.2)**	**0.7(0.5–0.9)**	**7.2(5.5–9.4)**	**9.1(8.5–9.8)**
**RSV**								
<6 months	35/279(12.5)	10.6(6.0–18.8)	13.4(7.5–23.8)	14.5(8.9–23.7)	2/72(2.8)	0.6(0.1–6.7)	6.5(0.6–71.4)	8.9(4.8–16.7)
6–11 months	39/337(11.6)	11.3(6.6–19.5)	14.0(8.1–24.1)	15.1(9.5–24.2)	14/128(10.9)	4.1(1.6–10.1)	40.6(16.4–100.6)	56.1(43.9–71.6)
12–23 months	49/409(12.0)	6.7(4.1–10.9)	8.1(5.0–13.1)	8.7(5.7–13.4)	21/223(9.4)	2.9(1.4–6.0)	29.0(13.8–60.8)	40.0(32.8–48.8)
2–4 years	37/319(11.6)	1.5(0.9–2.7)	2.0(1.1–3.4)	2.1(1.3–3.4)	49/402(12.2)	2.0(1.2–3.3)	19.7(12.1–32.0)	27.2(23.9–31.1)
5–17 years	7/126(5.6)	0.1(0.0–0.3)	0.1(0.0–0.4)	0.2(0.1–0.5)	13/221(5.9)	0.2(0.1–0.4)	1.5(0.6–3.8)	1.9(1.4–2.5)
18–34 years	7/117(6.0)	0.1(0.0–0.5)	0.1(0.0–0.5)	0.3(0.1–0.7)	1/35(2.9)	0.0(0.0–0.5)	0.2(0.0–4.9)	0.2(0.1–0.6)
35–49 years	1/67(1.5)	0.0(0.0–1.2)	0.0(0.0–1.3)	0.1(0.0–0.9)	*	*	*	*
≥50 years	1/76(1.3)	0.0(0.0–1.0)	0.0(0.0–1.1)	0.1(0.0–0.7)	*	*	*	*
<5 years	160/1344(11.9)	4.2(3.2–5.5)	5.2(4.0–6.8)	5.6(4.5–7.1)	86/825(10.4)	2.2(1.6–3.2)	22.3(15.5–32.2)	30.8(27.9–34.0)
≥5 years	16/386(4.1)	0.1(0.0–0.2)	0.1(0.0–0.2)	0.2(0.1–0.3)	15/267(5.6)	0.1(0.0–0.2)	0.9(0.4–2.2)	1.1(0.9–1.4)
** All ages**	**176/1730(10.2)**	**0.8(0.6–1.0)**	**0.9(0.7–1.2)**	**1.6(1.4–2.0)**	**101/1092(9.2)**	**0.4(0.3–0.6)**	**4.7(3.3–6.6)**	**6.0(5.4–6.5)**

a Includes only data for residents of the HDSS area of Keremo Division (2009–2012); ^b^Adjusted for those that met the case definition for SARI/ILI without laboratory test results; ^c^Adjusted for persons with pneumonia who did not seek care, using the results of a 2005 HUS; 48% (95% CI 35–62) of children <5 years and 34% (95% CI 23–48) of persons ≥5 years sought care for pneumonia at a hospital (Burton et al, 2005); ^d^Adjusted for persons with ILI who did not seek care, using the results of a 2005 HUS; 42%(95% CI 33–51) of children <5 years and 44%(95% CI 40–53) of persons ≥5 years sought care at any facility for ARI (Burton et al, 2005).

* Estimates not calculated because there were fewer than 30 specimens tested in this age-specific stratum.

Incidence rates of RSV-associated hospitalized SARI were 0.9 (95% CI 0.7–1.2) and were higher in children aged <5 years [5.2 (95% CI 4.0–6.8)] than in persons aged ≥5 years [0.1 (95% CI 0.0–0.2)] (Table 3). RSV-associated hospitalizations were highest in children <2 years old [13.4(95% CI 7.5–23.8) in children <6 months; 14.0(95% CI 8.1–24.1) in children aged 6–11 months; and 8.1(95% CI 5.0–13.1) in children aged 12–23 months]. As was the case with influenza, incidence of RSV-associated SARI was not significantly different in residents of Karemo living within 5 KM of SDH [<5 years = 4.6(95% CI 3.0–7.2); ≥5 years = 0.2(95% CI 0.1–0.5)] compared with those living within 10 KM of SDH [<5 years = 4.9(95% CI 3.7–6.6); ≥5 years = 0.1(95% CI 0.0–0.3)] and compared to those with 20 KM of SDH [<5 years = 5.2(95% CI 4.0–6.8); ≥5 years = 0.1(95% CI 0.0–0.2)] (Figure 4). More detailed age-specific incidence rates of hospitalizations associated with influenza and RSV are presented in Table 3. Incidence rates of hospitalizations associated with influenza or RSV were higher in the first year (August 2009– July 2010) compared to the other two years included in this analysis (Table 4).

**Table 4:** Annual rates^a^ for hospitalizations and outpatient visits attributable to influenza and RSV by year (per 1,000 persons) in Western Kenya, Aug 2009– Jul 2012.

	SARI				ILI			
	Year 1^b^	Year 2^c^	Year 3^d^	Average annual rates	Year 1^b^	Year 2^c^	Year 3^d^	Average annual rates
**Influenza A or B**								
<5 years	3.8(2.8–5.1)	3.2(2.2–4.5)	1.1(0.6–2.0)	2.7(1.8–3.9)	32.9(24.4–44.4)	15.3(9.9–23.7)	17.6(11.5–26.9)	21.8(15.1–31.6)
≥5 years	0.3(0.2–0.5)	0.2(0.1–0.4)	0.3(0.2–0.4)	0.3(0.2–0.4)	8.5(6.4–11.4)	3.1(1.9–5.0)	1.5(0.8–2.9)	4.3(2.8–6.4)
** All ages**	**0.9(0.7–1.1)**	**0.7(0.5–1.0)**	**0.4(0.3–0.6)**	**0.7(0.5–0.9)**	**12.7(10.3–15.6)**	**5.0(3.6–6.9)**	**4.1(2.9–5.9)**	**7.2(5.5–9.4)**
**RSV**								
<5 years	8.8(7.2–10.8)	4.8(3.6–6.4)	1.8(1.1–2.9)	5.2(4.0–6.8)	42.9(33.0–55.7)	13.8(8.7–21.9)	10.0(5.7–17.7)	22.3(15.5–32.2)
≥5 years	0.2(0.1–0.4)	0.1(0.0–0.2)	0.1(0.0–0.2)	0.1(0.0–0.2)	2.4(1.4–4.1)	0.2(0.0–1.4)	0.2(0.0–1.2)	0.9(0.4–2.2)
** All ages**	**1.7(1.4–2.0)**	**0.8(0.6–1.1)**	**0.3(0.2–0.5)**	**0.9(0.7–1.2)**	**9.8(7.8–12.5)**	**2.6(1.7–4.1)**	**1.8(1.0–3.1)**	**4.7(3.3–6.6)**

a Adjusted for those that met the case definition for SARI/ILI without laboratory test results;^ b^August 2009– July 2010; ^c^August 2010– July 2011; ^d^August 2011– July 2012.

### Incidence of medically-attended outpatient ILI and influenza and RSV-associated ILI

The estimated incidence of medically-attended outpatient visits associated with ILI among children <5 years of age was 214.9(95% CI 207.1–223.1), and 16.1(95% CI 15.1–17.1) among persons aged ≥5 years cases per 1000 persons (Table 2). The average annual adjusted incidence of medically-attended ILI associated with influenza was 7.2 (95% CI 5.5–9.4) and was significantly higher in children aged <5 years [21.8(95% CI 15.1–31.6)] compared to those aged ≥5 years [4.3(95% CI2.8–6.4)]. The average annual incidence of medically-attended ILI associated with RSV was 4.7 (95% CI 3.3–6.6) and was significantly higher among children <5 years [22.3(95% CI15.5–32.2)] compared to persons aged ≥5 years [0.9(95% CI 0.4–2.2)] (Table 2). The age-specific incidence rates of outpatient visits associated with influenza and RSV are presented on Table 3.

### Non-medically attended incidence rates of influenza and RSV SARI and ILI

The incidence of non-hospitalized SARI attributable to influenza was 1.2 (95% CI 0.9–1.4) [2.9 (95% CI 2.1–4.0) in persons <5 years; and 0.5 (95% CI 0.4–0.7) in persons ≥5 years]. The incidence of non-hospitalized SARI attributable to RSV was 1.6 (95% CI 1.4–2.0) [5.6 (95% CI 4.5–7.1) in persons <5 years; and 0.2 (95% CI 0.1–0.3) in persons ≥5 years] after adjusting for persons who reported cases of pneumonia but did not go to hospital.

The overall incidence of non-medically attended ILI attributable to influenza was 9.1 (95% CI 8.5–9.8); 30.1(95% CI27.3–33.3) in persons <5 years; and 5.4(95% CI4.9–6.0) in persons ≥5 years. The incidence of non-medically attended ILI associated with RSV was 6.0 (95% CI 5.4–6.5) [30.8(95% CI27.9–34.0) in persons <5 years; and 1.1(95% CI0.9–1.4) in persons ≥5 years] after adjusting for persons who reported ARI in the 2005 health utilization survey but did not go to any facility (Table 3).

## Discussion

These findings suggest an important burden of both influenza and RSV among persons with hospitalized SARI and outpatient ILI in western Kenya, particularly among children aged <5 years. There is also likely an important burden of illness associated with these viral pathogens in persons with SARI and ILI who do not seek medical care.

The burden of severe respiratory disease associated with both influenza and RSV was highest in children less than 23 months of age, suggesting young children are an important potential target group for current or future vaccines and other interventions in Kenya. While pregnant women are a high risk group for influenza-associated complications in their own right [25], our additional finding that the burden of influenza associated SARI was highest in those under six months of age further suggests the potential value of vaccination of pregnant women as a way of protecting (via maternal antibody transfer) those very young children for whom no influenza vaccine is currently licensed [26]. These data therefore support the 2012 recommendations by the WHO Strategic Advisory Group of Experts on Immunization that suggest pregnant women to be a high priority group for influenza vaccination [27].

The rates of influenza-associated hospitalizations presented here are comparable to the incidence reported in Kenya during the period August 2010 to July 2011 [11]. These rates of hospitalized influenza are also comparable to rates published for other locations in Kenya [11], and to those reported in Bangladesh and Thailand [28], [29]. However these estimates of influenza-associated hospitalizations in children <5 years are nearly two times higher than rates in the pre-pandemic period before 2009 in the same region [13]. Importantly, they are also 3–7 times higher than those reported recently in the United States [30], [31].

Our adjusted incidence of hospitalizations associated with RSV among children <5 years [i.e. 5.2 (95% CI 4.0–6.8)] is nearly 2-fold higher than the incidence reported in Kilifi, in the Coastal part of Kenya [14], but is lower than rates published in a five-year cohort study in South Africa. Even without adjustment, our observed incidence rates of RSV in children under 24 months of age also appear to be nearly twice those reported in the United States [32]. We also observe that the incidence of RSV-associated hospitalizations was higher than the incidence of influenza hospitalizations in younger ages, but lower in older ages, which is consistent with findings from other studies [31], [33].

Our observed incidence of outpatient ILI-associated influenza was only one-tenth of the rate reported in Bangladesh [28] but over 2-fold higher than rates reported in England and Netherlands among children <5 years [34]. As was the case with influenza, our estimated incidence of ILI-associated RSV in children <5 years was over 5-times higher than the incidence published in Europe [34].

Using adjustments for health seeking behaviors, our estimates of the incidence of non-medically-attended SARI and ILI associated with influenza and RSV were higher than comparable rates of medically-attended illness in this community. This highlights the need to consider burden of disease beyond the health care facility to best inform public health policy in this setting. Indeed, the 2005 survey indicated that only 48% of children <5 years and 34% of persons ≥5 years who reported having pneumonia sought care at a hospital. Similarly, only 42% of children <5 years and 44% of persons ≥5 years who reported having ARI in the two weeks preceding the health utilization survey sought healthcare at any health facility [21]. These direct estimates therefore support international indirect modeling efforts that have suggested influenza and RSV-associated severe disease rates are substantially higher in Africa and other lower resourced countries than elsewhere [2], [3], [5].

Our study was subject to several limitations. The SARI case definition did not include wheezing, nasal flaring, tachypnea, shortness of breath, and lethargy which have been associated with RSV elsewhere, and may have resulted in an under estimation of incidence of RSV, especially in very young children [3], [16], [35]. This might explain why our RSV rates were similar between infants aged <6 months and 6–11 months, which is inconsistent with other studies that report much higher rates in younger infants [32]. However, the SARI case definition that we used is broader than the new case definition recommended by WHO which requires a history of fever or measured fever [24]. There were an additional 30% SARI cases identified using this case definition compared to what would have been identified using the new WHO case definition. This may have served to improve sensitivity and thus helped to better estimate disease burden, in the very young children (<6 months) and older persons who may be less likely to present with fever as a component of influenza virus infections. The ILI case definition – requiring a measured temperature of ≥38°C – was used to estimate the burden of RSV among outpatients. This also, as the case with SARI, may have possibly led to an under estimation of the burden of RSV in outpatients, which may be less likely than influenza to result in presentation with fever [36], [37]. The case definitions for ARI and pneumonia in the 2005 health utilization survey were also both narrower in scope than the ILI and SARI case definitions used in facility-based surveillance, respectively. This could have led to an underestimation of the incidence of illness in the community.

Another limitation was that we used adjustment factors from a health utilization survey that was conducted in 2005– four years earlier than the study period – to estimate the burden of non-hospitalized and non-medically-attended illness. Health seeking behaviors could have changed over that period of time. Additionally, the survey was powered to estimate health utilization among for both children <5 years and persons ≥5 years [21]. In this respect, the health utilization adjustments may not have been as robust as ideal for the finer age-specific rate adjustments that we undertook. For example, we applied the same adjustment factor reported for health utilization among under-fives for all age categories in children <5 years (<6 months, 6–11 months, 12–23 months, 2–4 years). If the utilization rates differ across these age groups, then this could bias our estimates. Conducting health utilizations surveys with sufficient power in narrower age groups would serve to improve future estimates.

There was also minimal testing for HIV especially among younger persons and thus our study was not able to effectively estimate incidence specifically in HIV positive and negative populations. Lastly, we did not conduct a record review at hospitals in the area other than SDH to determine the proportion of residents of Karemo who went to other hospitals. Instead, we assumed that every resident of Karemo who required hospitalization would go to SDH. We felt this was a reasonable approach given that the next nearest hospital was located 17 KM away. To support this there were also no observed differences between calculated incidence for persons who lived within 5 KM, 10 KM, and 20 KM of SDH. The assumption behind this validation approach was that people within very close proximity would seek care at SDH rather than elsewhere and so we would have expected the incidence to decline significantly with an increase in distance.

In conclusion, influenza and RSV both exact an important disease burden in rural western Kenya, particularly in children <5 years old. These data suggest that a future RSV vaccine could have important implications for child health in Kenya. The immunization of children and pregnant women with currently licensed influenza vaccines also has important potential to reduce a significant cause of respiratory morbidity and mortality in Kenya.
